# Subtle predictive movements reveal actions regardless of social context

**DOI:** 10.1167/19.7.16

**Published:** 2019-07-29

**Authors:** Emalie G. McMahon, Charles Y. Zheng, Francisco Pereira, Ray Gonzalez, Leslie G. Ungerleider, Maryam Vaziri-Pashkam

**Affiliations:** emaliemcmahon@gmail.com; charles.zheng@nih.gov; francisco.pereira@nih.gov; raygon@mit.edu; ungerlel@mail.nih.gov; maryam.vaziri-pashkam@nih.gov; Section on Neurocircuitry, Laboratory of Brain and Cognition, National Institute of Mental Health, National Institutes of Health, Bethesda, MD, USA; Machine Learning Team, National Institute of Mental Health, National Institutes of Health, Bethesda, MD, USA; Machine Learning Team, National Institute of Mental Health, National Institutes of Health, Bethesda, MD, USA; Vision Laboratory, Department of Psychology, Harvard University, Cambridge, MA, USA; Section on Neurocircuitry, Laboratory of Brain and Cognition, National Institute of Mental Health, National Institutes of Health, Bethesda, MD, USA; Section on Neurocircuitry, Laboratory of Brain and Cognition, National Institute of Mental Health, National Institutes of Health, Bethesda, MD, USA

**Keywords:** action prediction, machine learning, psychophysics

## Abstract

Humans have a remarkable ability to predict the actions of others. To address what information enables this prediction and how the information is modulated by social context, we used videos collected during an interactive reaching game. Two participants (an “initiator” and a “responder”) sat on either side of a plexiglass screen on which two targets were affixed. The initiator was directed to tap one of the two targets, and the responder had to either beat the initiator to the target (competition) or arrive at the same time (cooperation). In a psychophysics experiment, new observers predicted the direction of the initiators' reach from brief clips, which were clipped relative to when the initiator began reaching. A machine learning classifier performed the same task. Both humans and the classifier were able to determine the direction of movement before the finger lift-off in both social conditions. Further, using an information mapping technique, the relevant information was found to be distributed throughout the body of the initiator in both social conditions. Our results indicate that we reveal our intentions during cooperation, in which communicating the future course of actions is beneficial, and also during competition despite the social motivation to reveal less information.

## Introduction

Walking down a busy street, remarkably, does not result in many people bumping into one another. When someone is walking directly toward you, you seamlessly predict the result of their action—a collision—and almost effortlessly adjust your path to pass by them. Similar predictions of actions are made when the barista hands you a coffee, or you shake a colleague's hand in greeting. Our ability to predict actions is essential in many social interactions (Frith & Frith, 2006), and may arise from the knowledge of biomechanical constraints of human movements (Johansson, 1973). In this paper, we would like to answer the following questions: What parts of the body reveal our actions to others? Do the informative regions of the body change through time? Does our ability to predict the actions of others vary depending on social context? Finally, do our social motivations change the availability of informative cues to others?

Previous studies have demonstrated humans' predictive ability when viewing the actions of others. For simple reaching actions, typical viewers have been shown to be able to predict the location of a movement's target before the movement was completed (Louis-Dam, Orliaguet, & Coello, 1999; Martel, Bidet-Ildei, & Coello, 2010; Pesquita, Chapman, & Enns, 2016; Vaziri-Pashkam, Cormiea, & Nakayama, 2017), and eye movements have been shown to follow the predicted direction of movement (Ambrosini, Pezzulo, & Costantini, 2015; Elsner, Falck-Ytter, & Gredebäck, 2012; Flanagan & Johansson, 2003; Flanagan, Rotman, Reichelt, & Johansson, 2013; Rotman, Troje, Johansson, & Flanagan, 2006). In more complex interactions such as competitive sports, it has been shown that humans and especially expert athletes can predict the consequence of actions in the physical world (Abernethy, Gill, Parks, & Packer, 2001; Abernethy & Zawi, 2007; Aglioti, Cesari, Romani, & Urgesi, 2008; Diaz, Fajen, & Phillips, 2012; Knoblich & Flach, 2001; Muller, Abernathy, & Farrow, 2006; Ranganathan & Carlton, 2007); for example, they can predict the direction of a soccer ball after viewing a videoclip (Diaz et al., 2012). Further, it has been suggested that the information enabling action prediction arises from kinematic features of the movement (Cavallo, Koul, Ansuini, Capozzi, & Becchio, 2016).

In a recent study (Vaziri-Pashkam et al., 2017), we investigated how humans predict the actions of others in a competitive reaching game. Participants responded remarkably fast relative to when their partner began reaching, suggesting that visual information was available prior to the explicit beginning of the movement (the lift-off of the finger from the starting point). Indeed, reaction times increased markedly (by around 100 ms) when predictive information prior to the start of movement was removed. Further, the information was not isolated to a single body part, as occluding large nonoverlapping sections of the body did not reduce reaction times. These results indicate the use of distributed predictive cues to actions, but they do not reveal the full spatiotemporal profile of the predictive cues, as the occlusion of information in space and time was done crudely. Here, using a novel machine learning analysis on videos of reaching actions combined with a psychophysics experiment, we aimed to more precisely determine the spatiotemporal profile of predictive kinematic cues to actions.

Using this combined approach, we also addressed whether the profile differs according to the social context. There is good reason to believe that social context might affect the availability of informative cues to actions. For instance, a forward in soccer does not want the goalie to know where she is directing the soccer ball. On the other hand, when passing the ball to her teammates, there is incentive to communicate the goal of her action. Here, the task demands—passing versus scoring—make it such that the actor clearly has different motivations in each context. Is the amount of information enabling action prediction modulated by the social motives of the actor? Perhaps this is the case given that a few studies have shown that, when looking at videos of actions, observers can determine if the action is intended to be cooperative or competitive (Manera, Becchio, Cavallo, Sartori, & Castiello, 2011; Sartori, Becchio, & Castiello, 2011). These studies and others indicate differences in movement kinematics between cooperation and competition (Becchio, Sartori, Bulgheroni, & Castiello, 2008; Georgious, Becchio, Glover, & Castiello, 2007), but, to the best of our knowledge, no one has directly investigated whether the availability of information is influenced by the social context.

**Figure 1:**
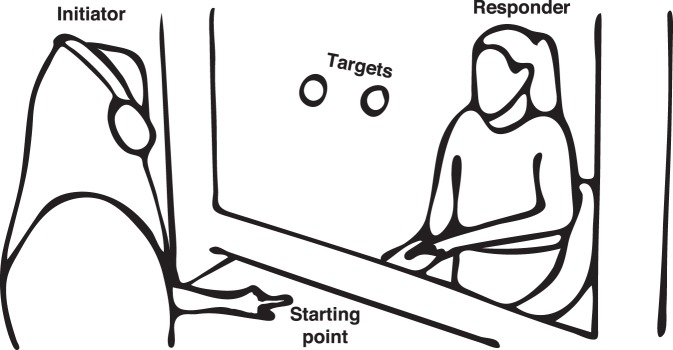
A schematic of the set-up of the game. The initiator (in headphones nearest viewer) was directed to touch either the left or the right target (circles in the center). The responder (opposite the initiator) was instructed to touch the same target at the same time as the initiator in the cooperative condition or beat the initiator to the target in the competitive condition.

To address this open question, we filmed participants in two reaching games that differed only in their social context, while all other task parameters were kept the same. In the game, two partners sat across from each other separated by a plexiglass screen with two targets on the screen. In the competitive game (analogous to the forward kicking the ball to score a goal), an actor (who we have termed the “initiator”) was signaled through headphones to touch one of two targets (Figure 1). The partner (called the “responder”) was asked to beat the initiator to the target. In the cooperative game (analogous to passing the ball to a teammate), the initiator also was signaled to touch one of two targets, but the two partners were told that the goal was to reach the target at the same time. From videos of initiators in this task, we first investigated when predictive information about actions became available to human observers. We then aimed to investigate where in the body these information cues originated and how this distribution changes over time. Because these are questions that would be difficult to answer precisely with human observers, we used information mapping by linearly decoding the direction of movement for each pixel in the videos (Kriegeskorte, Goebel, & Bandettini, 2006). These maps reveal what motion information is potentially available to human observers as they perform the same judgements. Finally, combining our analysis on human behavior and our classification results, we investigated how social context affected the distribution of the available information.

## Method

### Participants

#### Video collection

Forty-six adults (25 females, 21 males; *M* = 23.85 years, *SD* = 3.98 years) participated in the video recording phase of the experiment in which they were filmed playing a reaching game. The study was approved by the Harvard University Department of Psychology Institutional Review Board (IRB). All participants were right-handed and had normal or corrected-to-normal vision. All participants gave informed consent prior to study participation. Twelve pairs participated in the cooperative condition, and eleven participated in the competitive condition. One pair of participants in both conditions was excluded due to atypical movements by the initiator in one case, or experimental error in the other, and a random pair was removed from the cooperative condition to balance the number of participants in the two conditions.

#### Main experiment

Twenty participants (14 females, six males; *M* = 23.05 years, *SD* = 2.06 years) participated in the psychophysics experiment. The study was approved by the National Institute of Mental Health (NIMH) IRB. All participants included were in good health and free of psychiatric or neurological disease. Participants had normal or corrected-to-normal vision. All participants gave informed consent prior to study participation. Two participants were excluded after participation due to incidental findings in another study suggesting that they did not meet the healthy inclusion criteria specified in the IRB protocol, and one participant was removed due to poor performance (did not reach 75% accuracy even at the easiest time point). Thirteen of the participants took part in both the competitive and cooperative conditions, two only participated in the cooperative condition, and two only in the competitive condition. All participants watching videos of the initiators were unaware of the social context in which the videos were filmed. The final number of participants for each condition was fifteen (10 and 11 females in competitive and cooperative conditions, respectively).

### Procedure

The study consisted of two phases. In the first phase, videos and kinematic data were collected from participants in a cooperative or competitive reaching game. In the second phase, the videos were shown to separate human participants and used in machine learning analyses to determine the availability and spatiotemporal profile of predictive information across social contexts.

#### Phase 1: Collection of the videos and kinematic data

##### Videos

The initiators in the videos were recorded planning a reaching game with a partner. The initiators wore headphones and were signaled at the beginning of each trial to touch a left or right target with their right hand. The square targets (5 cm × 5 cm) were secured on a plexiglass screen (1.2 m × 1.5 m) separating the partners (∼1.2 m apart). Based on reaction times, a time window was set to be equal to the median hit time difference between the initiator and responder in the prior trials (except for the first five trials). In the competitive condition, the goal of the responders was to beat the initiator to the target. If the time difference between initiator and responder contact was smaller than the time window, the responder won; if it was larger, the initiator won. In the cooperative condition, the pair were told to hit the target at the same time. Both partners won if the time difference was less than the set time window, and both lost if it was greater.

Other than the terms to win, the only instructions given to the participants was to go directly to the target and not to trick their partner. At the end of each block, the total score for each participant was announced. The session occurred over five blocks of 30 trials. If there was an error during a given block, an additional block was added at the end. The game was recorded throughout using a Go-Pro camera positioned near the head of the responders to approximate their perspective. At the beginning of each trial, there was a green flash within view of the camera that allowed the videos of the blocks to be separated into individual trials (Vaziri-Pashkam et al., 2017).

The initiators in both conditions wore a Polhemus Liberty motion tracking sensor on the index finger of their right hand to identify the start of each trial and determine the velocity of movement. The position of the targets and the starting point of the initiator's finger was calibrated before the beginning of each session. After cutting the videos to individual trials, the spatial resolution of the videos was reduced to 675 × 380 pixels. The starting point was identified for each trial individually. Any trial in which the initiator made erroneous movements, began the movement by going to the incorrect target, or any other unusual occurrence (such as an experimenter walking into the frame) was not used in the psychophysics or machine learning phases of the experiment.

##### Kinematics

To determine the movement kinematics of the initiators in the videos, motion tracking sensors were attached to their index finger, which recorded the finger position at a rate of 120 Hz. The sensors were used to identify the beginning of the movement, the velocity of the reach, and how long the initiator took to reach the target from lift-off. The start point was defined as the moment in time when the velocity exceeded 20 cm/s. This velocity threshold was low enough that identification of the start point would not be biased by differences in velocity between the two conditions. All trials in which the initiator touched the incorrect target were considered inaccurate and were excluded from further analysis (<1% of trials). Further, because the initiators occasionally made small, erroneous movements, if the identified lift-off point was less than 50 ms from the beginning of the trial, the trial was excluded (<1% of trials). The average velocity in each trial was found by averaging the velocity from the start point to the touch point. The movement time was calculated as the difference in time between the lift-off and contact. The reaction time was calculated as the time from the instructions to the finger's lift-off. All trials were visually inspected to ensure that the lift-off and contact times were determined correctly. The average velocity, error rate, and reaction times were compared between cooperation and competition using an independent-samples, two-tailed *t* test.

#### Phase 2: Psychophysics and machine learning

##### Psychophysics

**Figure 2:**
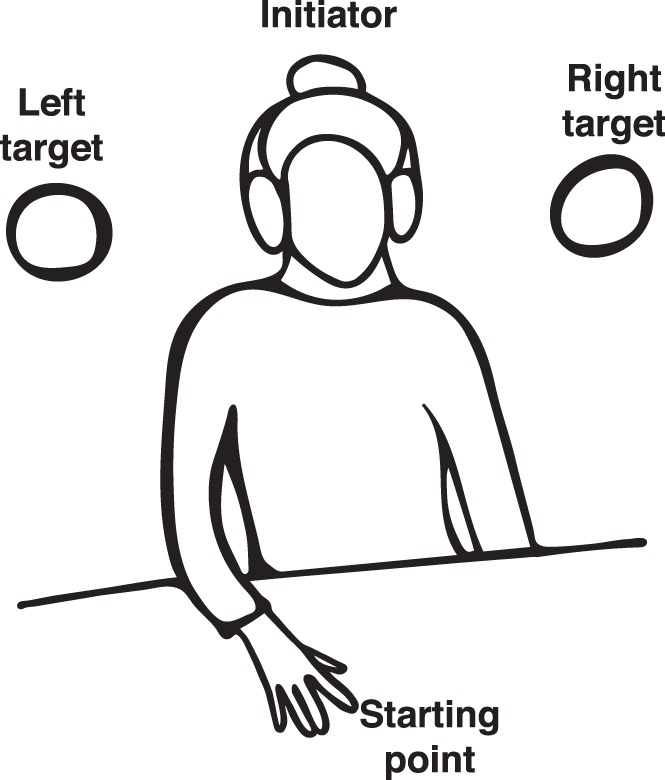
A schematic of a video frame presented to the participants in the psychophysics experiment. The video frames were presented in the center of the screen. The participants were asked to determine whether the person in the video was going to touch the left or the right target (relative to the viewer's perspective) with restricted information.

We ran a psychophysics experiment to determine when enough information is available in the early movements of the initiators to predict the direction of movement. Participants watched brief clips (example schematic of a video frame is depicted in Figure 2) presented on a black background and were asked to respond whether the initiator in the video was pointing towards the left or the right target (relative to their perspective) by responding on a keyboard with the index or middle finger of their right hand, respectively. The videos were displayed on a desktop monitor (BENQ XL) with a refresh rate of 60 Hz and spatial resolution of 1920 × 1080 from a MacBook Air using MATLAB (MathWorks, Natick, MA) and Psychtoolbox (Brainard, 1997). The stimuli were presented at a size of 10.4° × 5.9° of visual angle.

Cooperative and competitive conditions always occurred in separate experimental sessions because the experiment was long, and we wanted to ensure a complete set of data for at least one condition from each participant. Each session included 10 blocks. In each block only one initiator was shown. Because of the differencs in the body size of the initiators, randomizing initiators within block would have been distracting. Thus, the order of the blocks was randomized across participants. In each trial, a video clip was presented from ∼500 ms (∼30 frames) prior to the initiator start point (defined as the moment in time when the velocity exceeded 20 cm/s) to a cut frame that varied between 133 ms (eight frames) before the initiator's finger lifted off the table and 33 ms (two frames) after the finger lift-off. The final displayed frame remained on the screen for an additional 100 ms. This was to reduce possible masking of the final frame and allow for equally clear perception of all frames. Thus, the total presentation time ranged between 467 ms and 633 ms. Each of the possible cut frames (six cut frames) for the left and right direction (two directions) was repeated 10 times so that each block had a total of 120 trials. Between trials, a black screen was presented for at least 1.5 s or until a response occurred. Each trial presented was randomly selected from ∼70 possible video clips for a given initiator and direction.

This method yielded an average performance for each of the participants, initiators, and cut frames in both the cooperative and competitive social contexts. Performance was tested against chance at each of the six cut frames to determine when participants were able to predict the target of the initiator's reach. Because there were two sources of variance (the initiator and the participant), performance was tested against chance accuracy (50%) using a linear mixed model with both initiator and participant modeled as random factors (Kuznetsova, Brockhoff, & Christensen, 2017). Significance values were corrected for multiple comparisons using the Benjamini-Hochberg procedure for false discovery rate (FDR, Benjamini & Hochberg, 1995) implemented in MATLAB (Groppe, Urbach, & Kutas, 2011).

For each participant, the average accuracy at each cut frame for each initiator was determined. The accuracy by cut frame approximated a sigmoid and was fit with a logistic function using nonlinear least squares. Next, for each participant and each initiator, the time at which 75% accuracy was achieved (T_75_) was determined from the fitted function. Due to variations in performance, not all of the fits were ideal. Therefore, the T_75_ values that were found to be more than four median absolute deviations away from the median of the data (2% for cooperation and 2% for competition from the behavioral fits) were excluded from further analysis.

First, in a control analysis, to ascertain that the order of presentation of the participants in the block did not affect the results, the effect of order on performance (T_75_) was tested using a linear mixed model in which the order of initiator presentations (the blocks) was modeled as a fixed factor and participant was modeled as a random factor. Next, using a linear mixed model with participant and initiators as random factors, the main effect of condition was determined.

##### Machine learning

To further investigate when predictive information becomes available, a linear SVM was used to classify the direction of movement from the local motion information in the videos. Video frames were analyzed using Python's OpenCV (Bradski, 2000) Gunnar-Farnebäck optical-flow algorithm (Farnebäck, 2003) to calculate motion energy in the *x* and *y* directions for individual pixels between two subsequent video frames. In order to increase computational efficiency for classification, a movement filter was made by averaging the movement across *x* and *y* directions for all frames, trials, and participants; a motion energy threshold was set, and the filter was further manually cleaned to remove small, scattered clusters of pixels outside the body of the initiators. Note that the filter only selected pixels based on the overall amount of movement and not on the direction of movement. Therefore, the use of this filter should not have biased classification results. The resolution of the optical flow output and the filter were then down-sampled tenfold to further increase efficiency. The *x* and *y* direction optical-flow data were concatenated for this analysis. The analysis was done using LibSVM software (Chang & Lin, 2011) and custom MATLAB codes in a bootstrapped fashion: The SVM was trained on a random sub-sample containing 50% of the optical-flow data of one frame from nine videoed initiators and tested on the 10th, left-out initiator. This sampling was performed 100 times, which allowed us to estimate the confidence interval of the classifier's accuracy. This sampling, training, and testing method was repeated for all frames. The average accuracy for each frame was calculated across the 100 samples at each frame.

Similar to the behavioral data, the SVM accuracies were tested against chance at each frame using a linear mixed model in which the bootstrapping subsamples of the SVM were treated as different samples and modeled as a random factor. The initiator was also modeled as a random factor. Significance values were controlled for multiple comparisons using FDR correction (Benjamini & Hochberg, 1995).

To test how the information from one condition generalized to the other, a similar procedure was also used for cross-condition training and testing of the SVM. The SVM was trained on a random subsample containing 50% of the optical-flow data from all 10 initiators in one condition and a participant from the other condition. The random sampling was repeated 100 times, and this was done for each initiator. Similar to what had been done for the within-condition SVM, the classification accuracy of the cross-condition classifier was tested against chance accuracy using a linear mixed model with each subsample from the bootstrapping as a random factor as well as the initiator.

In the same way that had been done for the behavioral data, the SVM accuracies were fit with a logistic function using nonlinear least squares, and the T_75_ was determined. T_75_ outliers were removed if they were found to be more than four median absolute deviations away from the median of the data (0.1% for cooperation). The T_75_ of the SVM was also compared between cooperation and competition using a linear mixed model with the condition as the fixed factor and the initiator and the SVM bootstrapping subsample were both modeled as random factors.

Finally, the T_75_ was compared between humans and the SVM to determine whether one performed better than the other using a linear mixed model, with condition and SVM or human modeled as a fixed factor and participant and initiator as random factors. The SVM was averaged across the subsamples from bootstrapping before comparison.

Further, the T_75_ of the within- and cross-classification were compared using a linear mixed model in which testing method was modeled as a fixed factor and SVM bootstrapping sub-sample and initiator were modeled as random factors after averaging across cooperation and competition.

##### Considering the speed of movement

To determine how much of the difference between the cooperative and competitive conditions was due to the difference in velocity between the two conditions, the movement time of the initiator was considered. Because average velocity equals the distance traveled by the finger during the reach divided by the movement time, and because the distance between the starting location of the finger and the target was similar in all trials and between all initiators, in our case, using movement time is equivalent to using velocity, with shorter movement times indicating faster speeds. Thus, to take into account the velocity of the movement, in the linear mixed model for the human and the SVM performance in which T_75_ was compared between cooperation and competition, the movement time of the initiator determined from the kinematic data was added to the model as a fixed factor. This way, the effect of social condition could be considered in isolation from the effect of velocity.

##### Searchlight

The searchlight analysis was based on common practices in neuroimaging analysis (Kriegeskorte et al., 2006). Using the Searchmight Toolbox (Pereira & Botvinick, 2011) and in-house MATLAB codes, a Gaussian Naïve Bayes (GNB) classifier was trained to classify the direction of movement from the *x* and *y* optical-flow data within a neighborhood containing nine pixels around each pixel. We opted for a GNB classifier instead of an SVM classifier in this analysis to speed up the processing time.

The Searchmight provided an accuracy and *p*-value map for each initiator. The Searchmight Toolbox determines the *p* values against chance classification accuracy using a binomial test. These were corrected for multiple comparisons using FDR correction (Benjamini & Hochberg, 1995) and thresholded at FDR level *q* < 0.05. These first-level maps were then combined on the second level across initiators using a binomial test (see below for details). The final searchlight analyses were thresholded based on the second-level maps at FDR level *q* < 0.05.

In order to quantify differences in the distribution of information across the two conditions, the differences in the average maps for the two conditions were calculated, and this difference was tested against the null hypothesis of no difference using two-tailed bootstrapping *t* test over 10^5^ permutations for ∼5.9 × 10^4^ pixels. The resulting *p* values were corrected for multiple comparisons using FDR (Benjamini & Hochberg, 1995) correction.

To further determine whether the pattern of information in the body differed between the two conditions, we used a similar generalization method as used for the whole-frame classification analysis. The cross-condition searchlight was done by training on the data from one condition and testing on the data from one initiator for the other condition. This was done for every initiator, and the results averaged across them. The average classification accuracies were thresholded using the same first- and second-level procedure described previously.

To calculate the second level *p*-value maps in the searchlight analysis, we used a binomial test. For this, we individually thresholded the accuracy maps of each participant at an arbitrary false-discovery rate threshold, *q_1_*. We then computed the second-level *p* value for pixel *v* as

$$\newcommand{\bialpha}{\boldsymbol{\alpha}}\newcommand{\bibeta}{\boldsymbol{\beta}}\newcommand{\bigamma}{\boldsymbol{\gamma}}\newcommand{\bidelta}{\boldsymbol{\delta}}\newcommand{\bivarepsilon}{\boldsymbol{\varepsilon}}\newcommand{\bizeta}{\boldsymbol{\zeta}}\newcommand{\bieta}{\boldsymbol{\eta}}\newcommand{\bitheta}{\boldsymbol{\theta}}\newcommand{\biiota}{\boldsymbol{\iota}}\newcommand{\bikappa}{\boldsymbol{\kappa}}\newcommand{\bilambda}{\boldsymbol{\lambda}}\newcommand{\bimu}{\boldsymbol{\mu}}\newcommand{\binu}{\boldsymbol{\nu}}\newcommand{\bixi}{\boldsymbol{\xi}}\newcommand{\biomicron}{\boldsymbol{\micron}}\newcommand{\bipi}{\boldsymbol{\pi}}\newcommand{\birho}{\boldsymbol{\rho}}\newcommand{\bisigma}{\boldsymbol{\sigma}}\newcommand{\bitau}{\boldsymbol{\tau}}\newcommand{\biupsilon}{\boldsymbol{\upsilon}}\newcommand{\biphi}{\boldsymbol{\phi}}\newcommand{\bichi}{\boldsymbol{\chi}}\newcommand{\bipsi}{\boldsymbol{\psi}}\newcommand{\biomega}{\boldsymbol{\omega}}\Pr \left[ {{\rm{Binomial}}\left( {n,{{R{q_1}} \mathord{\left/ {\vphantom {{R{q_1}} {\left( {nV} \right)}}} \right. \kern-1.2pt} {\left( {nV} \right)}}} \right) \ge {c_v}} \right]{}$$

where *c_v_* denotes the number of participants for which that pixel was significant, *R* denotes the sum of the counts *c_v_* across all pixels. Under the null hypothesis for pixel *v*, the count *c_v_* has a binomial distribution with size *n* (the number of participants) and a probability that is approximately bounded by *Rq_1_*/(*nV*), where *V* is the number of pixels. After obtaining the second level *p*-value maps, all searchlight results were thresholded at false-discovery rate level *q* < 0.05.

## Results

### Availability of information predictive of the actions

**Figure 3:**
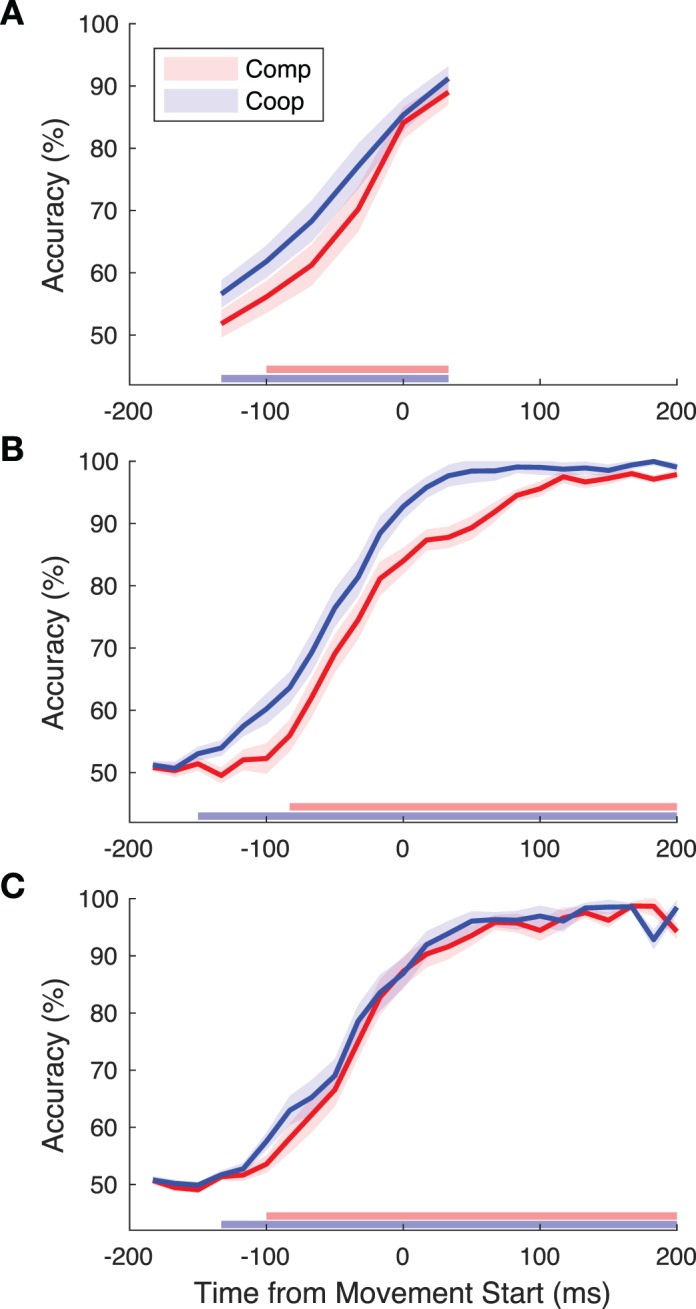
(A) Human participants and (B) within-condition and (C) between-condition SVM classifier accuracies for predicting the direction of the movement of the initiators in the videos. The accuracy for competition (red) and cooperation (blue) are compared to chance using a linear mixed-model regression at each time point. Time points in which the performance was significantly above chance are indicated near the x axis using a horizontal bar. The shaded areas on the curves depict the standard error of the mean obtained from the linear mixed models.

We aimed to determine whether both humans and a linear classifier could use early movement information to predict the action of an actor from videos. We showed video clips to participants not in the videos and asked them to determine which target the initiator in the video was reaching towards (Figure 2). The performance of the humans was then compared to chance using a linear mixed model. In the cooperative condition (Figure 3A), participants' performance was significantly above chance at all time points (all *t* > 3.0, all *p* < 0.007, corrected for multiple comparisons using false discovery rate for this analysis and all other tests reported in this section). In the competitive condition, performance was not above chance at the earliest frame (133 ms, *t* = 0.82, *p* = 0.42), but was significant at all subsequent time points (all *t* > 2.34, all *p* < 0.03). These results indicated that humans are able to determine the direction of a reaching movement prior to the explicit execution of the action, i.e., the finger lifting off from the table.

Next, for the same videos as those shown to participants, the motion energy between sequential pairs of frames was determined using a sparse optical-flow algorithm. An SVM was used to classify the direction of movement from the motion energy, and then the performance of the classifier was compared to chance using a linear mixed model at each frame of the video. The classifier (Figure 3B) reached above-chance classification accuracy at around 150 ms before the start of the movement in the cooperative condition (all *t* > 2.71 , all *p* < 0.02), whereas the accuracy before 150 ms was not above chance (all *t* < 1.81, all *p* > 0.09). In the competitive condition, the classification accuracy was significantly above chance beginning only 83 ms before the finger's lift-off (all *t* > 2.31, all *p* < 0.04). For all time points before 83 ms, performance was not significantly above chance (all *t* < 1.26, all *p* > 0.28). Together, the behavioral and SVM results indicate that there is considerable information available before the beginning of the finger movement in both cooperation and competition in accordance with previous work (Pesquita et al., 2016; Vaziri-Pashkam et al., 2017).

### Comparing human and the within-condition classification accuracies

**Figure 4:**
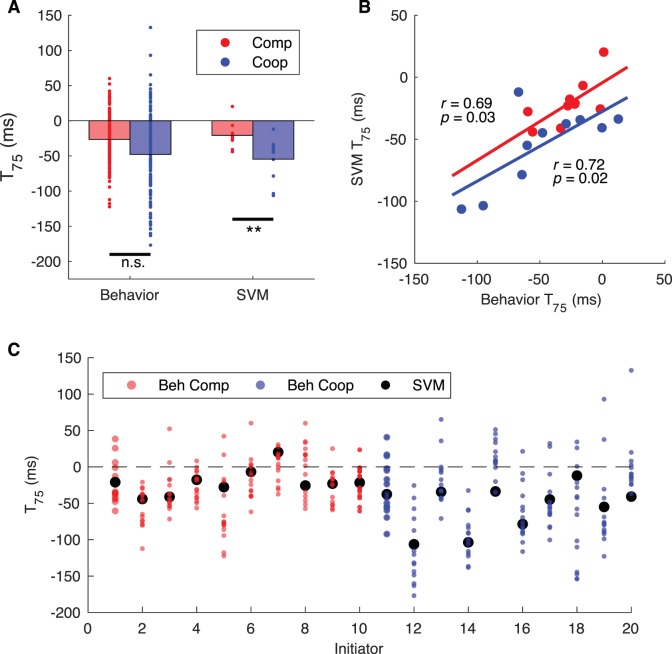
(A) Bars depict the mean time of 75% accuracy for humans and the within-condition SVM (n.s., p > 0.05, **p < 0.01). (B) The time of 75% accuracy for human performance correlated with the SVM time of 75% accuracy for competition and cooperation (C). There was no difference in the time of 75% accuracy for humans and the SVM.

The psychometric curve in the cooperative condition appears shifted to the left of the competitive curve (Figure 3A), suggesting that the actions may be revealed earlier in cooperation than competition. We fit the accuracy curves with a logistic function (see Method) and determined the time at which 75% accuracy (T_75_) was reached from the fitted curves. The T_75_ allowed us to summarize the curves with a single point and to quantify the differences between the two curves (the use of another time point, such as the time of 86% accuracy, did not significantly change the results). The T_75_ was compared between human and classifier and across social conditions using a linear mixed model (Figure 4).

The training and testing of the SVM was not perfectly analogous to human psychophysics. Humans saw videos of different initiators in different blocks with the two conditions presented in two separate sessions, while the SVM was trained on all data from nine initiators in each condition and tested on a left-out initiator. To ensure that the order of presentation did not have an effect on the human T_75_, using a linear mixed model with order of the presentation as a fixed factor and participant as a random factor, we found that there was no effect of order on human performance (*t* = 0.02, *p* = 0.99). In other words, no fatigue or training effect was observed.

No significant difference was found between the SVM and human T_75_ (*t* = 0.17, *p* = 0.87, Figure 4C), nor was there an interaction between the effect of SVM versus human and the social condition (*t* = −0.60, *p* = 0.55). Thus, there is no evidence that the SVM reached 75% accuracy at a different time than human viewers. In addition, the T_75_ of the SVM strongly correlated with the behavioral performance in both the competitive (*r*(8) = 0.69, *p* = 0.03) and cooperative (*r*(8) = 0.72, *p* = 0.02) conditions as well as overall (*r*(18) = 0.74, *p* < 0.001); see Figure 4B.

Combined across human and SVM, there was no significant effect of social condition (*t* = −1.84, *p* = 0.08). Looking at SVM and human performance independently, the T_75_ was found to be earlier in the cooperative condition than in the competitive condition for the SVM (*t* = −2.95, *p* = 0.009), whereas for humans, there was no main effect of condition (*t* = −1.79, *p* = 0.09), illustrating that the overall lack of an effect was strongly influenced by the behavioral results (Figure 4A).

These findings indicate that the SVM is able to pick up on predictive information earlier in the cooperative condition than the competitive condition, but we did not observe a similar effect in humans, likely due to greater variance in human performance. Further, the SVM performance was comparable to the performance of humans.

### Classification generalization analysis

Although there was no difference in human performance across social conditions, the classification results suggest that the target of the reach may become apparent earlier in cooperation than in competition. But, how similar is the available information in the two conditions? To further understand the differences and similarities between the cooperative and competitive conditions, we used a generalization analysis. In this analysis, we trained a classifier to discriminate the two movement directions in one social condition and tested it in the other social condition and vice-versa (cross-condition classification; see Method) and compared it to the results from the last section where the training and testing of the classifier was performed within the same condition (within-condition classification). The results should fall somewhere between two extremes. One possibility is that there are no shared informative features between the two conditions. If this were the case, the cross-condition classification accuracy at all time points should be at chance. The other possibility is that the informative features between the two conditions are exactly the same. In this case, we would expect the cross-condition classification accuracy to be identical to the within-condition classification accuracy.

What we found is that when we trained on the competitive data and tested on the cooperative (Figure 3C), the cross-condition classification reached above-chance accuracy 133 ms before the start of the movement (all *t* > 2.34, all *p* > 0.04). All points before 133 ms were not significantly above chance (all *t* < 1.17, all *p* > 0.28). The classifier reached above chance classification accuracy at 100 ms before the start when trained on the cooperative data and tested on competitive data (all *t* > 2.32, all *p* < 0.04), but not before 100 ms prior to the start (all *t* < 1.87, all *p* > 0.09). Averaging across social conditions, the cross-condition classifier reached 75% accuracy later than the within-condition classifier (*t* = 14.70, *p* < 0.001). Thus, the lower accuracy in the cross-condition classification compared to within-condition classification does hint at differences between the two conditions, but the early above-chance classification accuracy of the cross-condition classifier illustrates that there is considerable shared information between the two conditions.

### Considering the speed of movement

In the previous sections, we found that the SVM classifier can determine the direction of movement earlier in the cooperative than the competitive condition. How much of this difference between the T_75_ in the two conditions from the SVM analysis could be accounted for by the difference in the speed of movement between the two conditions? During the video collection phase of the experiment we also collected kinematic data from the finger of the initiators (see Method). Not surprisingly, we found that initiators moved more quickly in competition (*M* = 221.3 cm/s*; SD* = 42.9 cm/s) than cooperation (*M* = 114.0 cm/s*; SD* = 10.6 cm/s; *t*(18) = 7.68, *p* < 0.001) while their reaction times (*t*(18) = 0.27, *p* = 0.79) and accuracies (*t*(18) = −1.63, *p* = 0.12) were not significantly different between the two conditions. There was a significant positive relationship between the T_75_ and movement time, as determined by a linear mixed model for both the SVM (*t* = −3.11, *p* = 0.006) and humans (*t* = −2.59, *p* = 0.02), suggesting that the direction of movement of slower initiators can be determined earlier than faster initiators. To take speed into account in our analyses, the average finger traveling time (from the start of the finger lift-off to the time of the target hit) for each initiator was taken as a measure of movement speed and added to the linear models as a separate factor. The main effect of condition for the SVM was no longer significant (*t* = −0.39, *p* = 0.70). There was previously no difference in the behavioral data, but when factoring movement time into account, the distributions of the two conditions became more overlapping (*t* = 1.07, *p* = 0.30). These results, thus, suggest that cooperative initiators may move more slowly to allow their partners to process the information as it becomes available.

### Searchlight analysis

**Figure 5:**
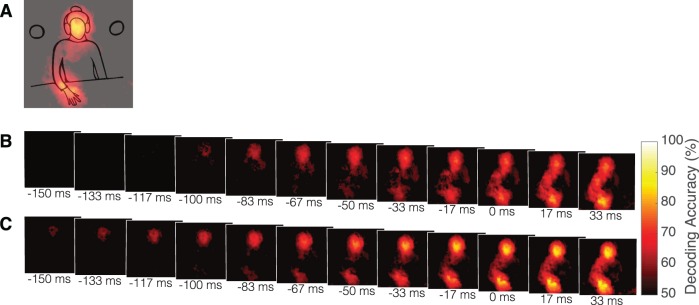
(A) From the cooperative condition, the frame at −17 ms is overlaid on the video schematic shown in Figure 2. The overlay provides an approximation for the body of the initiator relative to the classification accuracies reported. The same approximation is appropriate for all maps that follow. The average classification accuracy for the within-condition searchlight classifier through time for (B) competition and (C) cooperation where the accuracy is thresholded at the second level at q < 0.05.

Previous analyses indicate the existence of predictive cues to actions before finger liftoff and suggest that the information is, to some extent, similar regardless of the social motivations. To investigate more precisely the spatiotemporal profile of the informative cues, we borrowed a technique from neuroimaging (Kriegeskorte et al., 2006), namely, searchlight information mapping. By training and testing a GNB classifier within small regions of the video frame, we were able to determine whether each location in one frame of the optical-flow data of the initiators' body contains predictive information about their actions. The classification accuracies across time (averaged across the initiators and thresholded at *q* < 0.05) are shown in Figure 5B for competition and Figure 5C for cooperation. These results show that, for both social conditions, the information is widely distributed throughout the body of the initiator.

**Figure 6:**
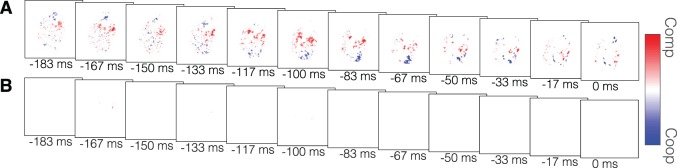
The average difference in classification accuracy at each point between cooperation and competition through time. Redder indicates those regions more informative in competition, while bluer indicates those more informative in cooperation. (A) Some pixels show differences before FDR correction within a motion-filtered region at p < 0.05, but (B) few pixels remain after FDR correction. Note: Panel B should be interpreted by the reader as the difference between the two conditions because the accuracy differences in Panel A are not controlled for multiple comparisons.

We also compared the cooperative and competitive conditions (see Method). The average difference is shown in Figure 6, both thresholded at *p* < 0.05, uncorrected (Figure 6A) and corrected for multiple comparisons using FDR and thresholded at FDR level *q* < 0.05 (Figure 6B). Interpreting Figure 6B, which shows the results after correction for multiple comparisons, this analysis shows that there was little difference between the distribution of information in the bodies of the cooperative and competitive initiators.

**Figure 7:**
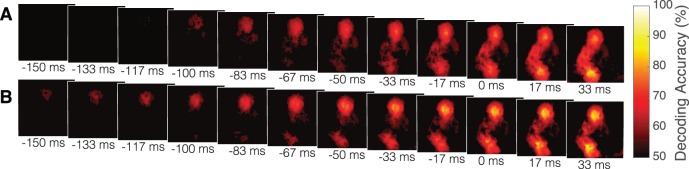
The average classification accuracy for the cross-condition searchlight classifier through time for (A) competition and (B) cooperation where the accuracy is thresholded at the second level at q < 0.05.

The searchlight analysis was also performed by training the classifier on the data from one condition and testing it on the other condition, similar to what was done for the whole frame cross-condition classification analysis. The results of this analysis (corrected for multiple comparisons at FDR level *q* < 0.05) are shown in Figure 7A for training on cooperation and testing on competition and vice-versa in Figure 7B. These results further confirm the conclusion that local information is similar in the two conditions.

Overall, the searchlight results indicate that information is distributed throughout the body of both cooperative and competitive initiators with little difference in the distribution of information between the two conditions.

## Discussion

We aimed to investigate the spatiotemporal profile of the cues people use to predict the actions of others and whether the social context affects the availability and distribution of these cues over the body. We found that people are able to predict the actions of others well before explicit movement execution. Further, in cooperation, the initiators reach more slowly to benefit their partner, but, aside from reaching more slowly, the available information is similar in cooperation and competition. Moreover, the information is distributed throughout the body, and the spatiotemporal profile of the information is similar across social contexts.

One contribution of this work is the combination of behavioral techniques as well as machine learning classifiers on videos of human actions to characterize when information becomes available during the course of a movement. Comparing the accuracy of humans in a behavioral experiment with the accuracy of a linear classifier, we found that both humans and the classifier were able to determine the direction of movement with above-chance accuracy as early as 150 ms before the finger had even lifted off the table to begin the reach. The accuracies were as high as 80% by the time the finger started to lift-off. These results are in line with our previous research (Vaziri-Pashkam et al., 2017, see also Pesquita et al., 2016), demonstrating that subtle cues in the body of humans reveal actions well in advance of the action execution. The previous study used a naturalistic task and showed that removing the early, predictive cues slows reaction times. Here, we showed videos of a similar task to participants and asked them to explicitly indicate the direction of movement. This allowed us to reveal more precisely the time at which the information becomes available. We found that when asked explicitly, participants are able to predict the direction of movement well before the initiator's finger has lifted off.

When we trained a linear classifier to perform the same task, notably, the classifier's performance was not significantly different from human performance. Thus, a simple algorithm based on instantaneous optical flow suffices to explain the findings we observed in human performance. This is surprising given that humans have ample experience observing the actions of others and likely have some prior knowledge about biomechanical limitations of the body and social expectations about how the initiator will behave. Further, the performance of the linear classifier also correlated with the performance of humans. In other words, if it was difficult for a human to determine the direction of movement of a given initiator, it was also difficult for the linear classifier to decode the direction of movement of the same initiator. What this correlation indicates is that whatever visual information humans use to determine the direction of movement may be tightly linked to the local optical-flow information. Despite this correlation, we cannot make any strong claims about the exact nature of the information used by humans; either local kinematic cues or global biomechanical information may be used. Future research looking at the sensitivity of human action prediction to different biological movement cues could shed light on this open question.

Beyond the limitations in conclusions to be drawn from comparisons of human and classifier performance, other limitations exist. There was a notable difference in the way in which the classifier was trained and the psychophysics experiment. Humans saw one subject at a time in a blocked-design, while the classifier was iteratively trained on nine initiators and tested on a single left-out initiator. To address if the order of presentation affected the psychophysics results, we established that there was no effect of video presentation order on performance. In other words, no significant fatigue or training effect was observed during the course of the psychophysics sessions. The lack of training effect is in line with our previous research in which, using similar videos, we found that subjects' performance did not change throughout the course of the experiment (Vaziri-Pashkam et al., 2017). The lack of training effect in the previous and current findings suggests that participants must be experts in predicting the actions of others prior to participation. Training the classifier in an analogous manner to the cumulative life experience of our participants is intractable. Thus, we opted for training the classifier on all initiators except one to increase the possibility that the classifier would generalize across initiators while maximizing the amount of training data. The effect of various training regimes on the classification results could be addressed in future studies.

Another difference between the classification analysis and the psychophysics experiment was that the classifier was trained and tested on a single frame of the optical-flow data, whereas humans were shown videos of around 500 ms. Even if humans were not integrating motion signals from the full ∼500 ms clip, it remains possible that they were integrating motion over multiple frames of the video. Ideally, we should have used multiple frames matching the videos humans saw as the input to the classifier. However, this procedure would have led to an input data structure with very high dimensionality without an increase in the number of features increasing the likelihood of overfitting the training data. We, therefore, opted for training the classifier on only a single frame of the optical-flow data. The effect of training the classifier on a biologically meaningful timescale remains an open question. However, the finding that a classifier trained on only a single frame of the optical flow is able to perform similarly to humans seeing a longer videoclip may suggest either that there is shared information between frames and/or that humans weigh the later frames of each clip (that contain the largest amount of information) higher than the earlier frames. Future studies are required to systematically address how the cues for action prediction are integrated across time.

Furthermore, this experiment utilized a linear SVM to discriminate the two movement directions. In contrast to nonlinear classifiers, the results of linear classifiers are more interpretable (Naselaris, Kay, Nishimoto, & Gallant, 2011). For instance, high performance of a linear classifier reveals that the data are linearly separable into classes. Moreover, a linear SVM is the best classifier for our data considering that the common alternative linear classifiers (logistic regression, linear discriminate analysis, and Gaussian Naïve Bayes classifiers) either have assumptions that may not be met by our data or are unsuitable for high-dimensional data (Hastie, Tibshirani, & Friedman, 2009). However, although a linear SVM was chosen due to the structure of our data, the results should not be interpreted as specific to a linear SVM. If the analysis were to be repeated with other linear classifiers, we expect similar results, which is often true in comparisons of different linear methods (Naselaris et al., 2011). Indeed, in our searchlight analysis, we used a GNB classifier because there were fewer features in each searchlight window, and we needed to speed up the process (SVMs are considerably slower than GNB classifiers) due to the large number of tests performed in the searchlight analysis.

Although there are fundamental differences between humans and machines, there are advantages in combining methodologies. Previous research, particularly in computer science, has had success in validating the performance of computer algorithms against the performance of humans. In this approach, human performance is a standard to be met and eventually surpassed by the algorithm. Object recognition is one example of success using this approach (He, Zhang, Ren, & Sun, 2015). Here, we have taken a different approach. Notably, we used a vanilla linear classifier rather than more complex algorithms such as a deep convolutional neural network, which may have reached a higher level of performance. This methodological decision was made because we aimed to use classification to uncover the true source of local information that may have been available to humans. Running a full psychophysics experiment equivalent to the searchlight analysis in humans would not be feasible. However, by uncovering the spatio-temporal profile of motion information using a machine learning algorithm, we can inform future psychophysics research to focus on specific body parts and time points to investigate how humans integrate the available information in space and time.

From this combination of analysis methods, we demonstrated that predictive cues to actions are widely distributed throughout the initiator's body. In a previous study, we suggested that this may be the case by selectively occluding different regions of the body and asking participants to predict the direction of movement (Vaziri-Pashkam et al., 2017; see also Pesquita et al., 2016). However, these measures were crude and unable to show the precise spatiotemporal profile of the informative body regions. Here, using a novel application of searchlight information mapping common in neuroimaging (Kriegeskorte et al., 2006), we were able to measure the spatiotemporal profile of the informative cues more precisely and comprehensively. This analysis confirmed that the information is widely distributed throughout the body of the initiators.

From the searchlight information mapping, we note that while the earliest information appears to be located in the head, the hand and wrist are added next, and then the information quickly becomes distributed throughout the body. Speculating on the source of the observed patterns in the SVM, initiators may first orient their attention to the directed target, which causes the head and the upper torso to reveal information. They may then begin to make small preparatory movements in the hand and wrist, adding additional sources of information. Next, as they prepare to make a large reach, they make stabilizing postural adjustments in other regions of the body to prepare for the shift in the center of gravity, causing the information to be distributed over larger regions of the arm and torso. It has been known for some time that such adjustments occur (Hodges, Cresswell, Daggfeldt, & Thorstensson, 2000; Hodges, Cresswell, & Thorstensson, 1999), but it was unknown until recently that these movements may be visually informative to others about the direction of a reaching action (Vaziri-Pashkam et al., 2017).

The searchlight results do indicate that information is distributed throughout the body of the initiators. However, the analysis does not show what information people are actually using to inform their decision. People may not be performing the task optimally by simultaneously integrating all the available information. For instance, they may attend to local regions of the body without integrating more diffuse information, or they may be attending to regions that do not seem to contain predictive information. The searchlight results should be interpreted as where local information is available and may be used in future research to constrain hypotheses on how humans may be performing the task.

Relatedly, our searchlight information mapping did not reveal the eyes to be particularly predictive. One outstanding possibility is that people may primarily rely on the gaze direction of the initiators to predict their actions. Although this seems to be an obvious source of information, we think that it is unlikely that gaze was a substantial source of information in our experiments. First, others have found that viewers are more sensitive to kinematic cues than to gaze (Quesque & Coello, 2014). Further, researchers who have used occlusion paradigms have shown that participants are able to perform the task even when the eyes are occluded (Pesquita et al., 2016; Vaziri-Pashkam et al., 2017). Thus, we do not think it likely that gaze is the most significant contributor to the ability of humans to predict the actions of others.

Investigating the effects of social context on the availability of predictive cues revealed by information mapping, we found that the classifier was able to predict the direction of movement of the cooperative initiators earlier than the competitive initiators. This contrast would seem to suggest that the cooperative initiators may be able to augment the information revealed by their movements to benefit their partner or, conversely, that the competitive initiators may reveal less information to their opponent. Contrasting the searchlight accuracies for the two conditions, we saw that there were only subtle differences without multiple comparisons correction, which disappeared after correction for multiple comparisons. Thus, overall, there was little significant difference in the distribution of information between the two social conditions. However, given the small number of participants and the large number of pixels, this analysis may not have enough power to detect small differences. Nevertheless, the high accuracies in the cross-condition whole-frame and cross-condition searchlight analysis showed that the classifier trained on the optical-flow information from one condition can predict the direction of movement in the other condition. This result strongly suggests that the information available in the two conditions is highly similar.

Yet, the difference in classification performance between the two conditions remains, which seems to contradict the searchlight findings. Note, however, that the whole-frame classification is more powerful in detecting differences between the two conditions, as it aggregates the information across all pixels. What could account for these conflicting results? One feature unambiguously different between cooperation and competition is the speed of movement. Perhaps in order to coordinate their actions (Sebanz, Bekkering, & Knoblich, 2006), the actors reach more slowly in cooperation. Similar speed differences have also been found by others (Becchio et al., 2008; Georgious et al., 2007; Manera et al., 2011; Sartori et al., 2011; but see Quesque, Mignon, & Coello, 2017 for evidence to the contrary). When the effect of movement time (increased movement time is associated with decreased T_75_) was taken into account in the analysis of the time of 75% accuracy, there was no longer a difference in the time that classification reached 75% accuracy between cooperation and competition. This analysis, combined with the fact that information becomes available earlier in cooperation than in competition, suggests that the cooperative and competitive initiators may be employing different strategies to achieve their different social goals. The competitive initiators reveal information later and reach quickly, compressing how much information is available in time. In contrast, the cooperative initiators begin revealing information earlier and reach more slowly, stretching the availability of information in time. This may afford their partners more time over which to accumulate information. This trend seems to be reflected in the searchlight maps. Although there were no significant differences after controlling for multiple comparisons, qualitatively, the maps do suggest that information becomes available earlier and is stretched in time in cooperation relative to competition.

Note, although there is a large difference in the velocity of the initiators in cooperation compared to competition, we did not find a difference in the accuracy or reaction time of the initiators in responding to the instructed target. Thus, while the initiators are modulating the speed of their movement based on the social motivations; they did not respond differently to the trial cue. In both conditions, only the initiators heard the cue, while the responders did not. The responders had no way of assessing the changes in the reaction time of the initiators without hearing the cue. Therefore, the initiators could not use a modulation of reaction time to either advantage or disadvantage the responders. Different results may be observed in a paradigm in which both partners are cued at the beginning of the trial.

Our study may seem at odds with previous studies showing that observers can discriminate cooperative from competitive movements in videos (Manera et al., 2011; Sartori et al., 2011). Note, however, that our design differs from these previous studies in that viewers of the movement were not asked to discriminate between cooperation and competition but, rather, simply to predict the intended direction of movements. Additionally, although previous studies kept the beginning of the actors' movements the same between cooperation and competition, the end of the motor sequence differed between the two conditions. Here, the action performed in both contexts was the same throughout. Moreover, our analysis considering the speed of movement suggests that the ability to discriminate between cooperation and competition may be explained by differences in speed.

Whereas we found predictive cues were present in the body of the competitive initiators, in the present study, we explicitly instructed participants in the competitive condition not to deceive their partner and to reach directly for one of two targets based on instructions. Yet, we know that deception is used to conceal intent in many different social interactions, such as sports and poker. Previous studies have investigated the detection of deception in sports, such as fake passes in basketball (Sebanz & Shiffrar, 2009). The results of our study suggest that the reason that humans use deception in competition is to distract their opponent from the relevant information betraying their action goals. Future investigations could reveal how deception modulates the availability of information in the body of the actor.

In addition to the lack of deception in our experiment, in further contrast to daily life in which most of actions are freely chosen, in the current study, the initiators were always instructed to reach to one of the two targets. It has been suggested that freely choosing an action may affect the predictive cues available to others (Pesquita et al., 2016). We suspect that our findings would be comparable if our initiators freely chose the target of their reach rather than being instructed, but future research is needed to establish the generalizability of our findings.

Finally, our results have implications for future models of action prediction. Researchers have previously proposed that to predict action goals humans either simulate the full course of actions (Flanagan & Johansson, 2003; Rizzolatti, Fogassi, & Gallese, 2001), infer the goal of the action from contextual knowledge (Brass, Schmitt, Spengler, & Gergely, 2007; Csibra, 2008), or some combination of the two (Ambrosini et al., 2015; Falck-Ytter, 2012). While we are unable to specifically support or refute any of these different models, we found that, first, the predictive cues to actions emerge well before the explicit part of the movement starts, and second, human performance is similar to and correlated with a linear classifier based on local motion information. Thus, even though we cannot directly address the underlying mechanisms of action prediction, our results suggest that, to predict actions, simulations or inferences should heavily weigh the early, subtle predictive movements and may have features that are linked to the local kinematic information.

In sum, our findings indicate that people are able to predict the actions of others even before explicit movement execution and that the availability of this information does not change across social contexts, even when those contexts demand such modulations. Determining whether these findings generalize to other settings could have implications for models of human action prediction and may inform the development of computer systems interacting in social settings.
